# *Penthorum chinense* Pursh Compound Ameliorates AFB1-Induced Oxidative Stress and Apoptosis via Modulation of Mitochondrial Pathways in Broiler Chicken Kidneys

**DOI:** 10.3389/fvets.2021.750937

**Published:** 2021-10-08

**Authors:** Weilai Tao, Zhenzhen Li, Fazul Nabi, Yu Hu, Zeyu Hu, Juan Liu

**Affiliations:** ^1^College of Veterinary Medicine, Southwest University, Chongqing, China; ^2^Chinese Veterinary Herbal Drugs Innovation Research Lab, University Veterinary Science Engineering Research Center in Chongqing, Chongqing, China; ^3^Immunology Research Center, Medical Research Institute, Southwest University, Chongqing, China

**Keywords:** *Penthorum chinense* Pursh compound, kidney, broilers, aflatoxin B1, apoptosis, autophagy, mitochondrial DNA

## Abstract

Aflatoxin B1 (AFB1) is a carcinogenic mycotoxin widely present in foods and animal feeds; it represents a great risk to human and animal health. The aim of this study was to investigate the protective effects of *Penthorum chinense* Pursh compound (PCPC) against AFB1-induced damage, oxidative stress, and apoptosis via mitochondrial pathways in kidney tissues of broilers. One-day-old chickens (*n* = 180) were randomly allocated to six groups: control, AFB1 (2.8 mg AFB1/kg feed), positive drug (10 mLYCHT/kg feed), and PCPC high, medium, and low-dose groups (15, 10, and 5 ml PCPC/kg feed, respectively). AFB1 treatment reduced weight gain and induced oxidative stress and kidney damage in broiler tissues; however, PCPC supplementation effectively enhanced broiler performance, ameliorated AFB1-induced oxidative stress, and inhibited apoptosis in the kidneys of broilers. The mRNA expression levels of mitochondria-related apoptosis genes (Bax, Bak, cytochrome *c*, caspase-9, and caspase-3) were significantly increased, whereas *BCL2* expression level decreased in the AFB1 group. Supplementation of PCPC to the AFB1 group significantly reversed the changes in mRNA expression levels of these apoptosis-associated genes compared to those in the AFB1 group. The mRNA levels of *NRF2* and *HMOX1* in the kidneys of the AFB1 group were significantly reduced compared to those in the control group, whereas PCPC significantly increased the *NRF2* and *HMOX1* mRNA levels. AFB1 decreased the levels of Beclin1, LC3-I, and LC3-II and increased P53 levels in the kidney compared to those in the control, whereas PCPC significantly reversed these changes to normal levels of autophagy-related genes compared to those in the AFB1 group. In conclusion, our findings demonstrated that PCPC ameliorated AFB1-induced oxidative stress by regulating the expression of apoptosis-related genes and mitochondrial pathways. Our results suggest that PCPC represents a natural and safe agent for preventing AFB1-induced injury and damage in broiler tissues.

## Introduction

Aflatoxins are carcinogenic mycotoxins produced by *Aspergillus* fungi, among which aflatoxin B1 (AFB1) is the most toxic and widely present in various foods and feeds and represents a serious risk to human and animal health (1, 2). AFB1 produced by moldy feed causes liver, kidney, and other organ damage and inhibits the immune function of the body which considerably affects the growth performance of livestock and poultry, thereby causing huge losses to the livestock and poultry breeding industry globally (3–5). Moreover, AFB1 and its metabolites (AFM1) may accumulate in meat products, which represents a serious risk to human health (6).

The kidneys are vital organs that perform many functions including removal of waste from the blood, regulation of the dynamic balance of water and electrolytes, and urine production (7–10). The absorbed mycotoxins are removed directly through urine and it is excreted in droppings in the poultry; however, the mycotoxin residues in the kidney pose animal and human threat, since the kidney cells are directly damaged by AFB1, and increase cell apoptosis and death. The liver and kidney are considered to be the main target organ of AFB1 with signs of liver injury, hepatic histological lesions, and renal atrophy (11, 12); however, recent studies have also shown that after consumption of feed rich in AFB1, the accumulation of AFB1 and AFM1 in the kidneys may cause damage to the kidneys in broiler chickens (*Gallus gallus domesticus*) (13). The toxic effects of AFB1 are mainly mediated via oxidative stress; AFB1 induces the formation of free radicals and inhibits the production of antioxidant enzymes, which leads to an imbalance between oxidation and anti-oxidation and increases oxidative damage (14).

Apoptosis involves programmed cell death, which plays an important role in maintaining normal tissue homeostasis. However, excessive apoptosis leads to the development of various diseases (15). AFB1, which accumulates in the kidneys, may induce kidney cell apoptosis via cytotoxicity (16). Autophagy is an efficient mechanism via which host cells resist cytotoxicity and it plays a crucial role in maintaining organism homeostasis in both physiological and pathological situations (17). Autophagy is closely related to apoptosis; therefore, AFB1 may exert toxic effects by inducing oxidative stress generation and cell apoptosis and by regulating autophagy in the chicken kidneys.

*Penthorum chinense* Pursh is a compound preparation (PCPC) composed of traditional Chinese herbal medicines such as *P. chinense* Pursh, *Bupleurum*, Yinchen, and licorice. The main ingredient *P*. *chinense* Pursh has antioxidant and anti-liver cancer activities (18), and may exert therapeutic effects and reduce adverse effects via the synergistic effect of multiple active ingredients from different plant parts (19–21). PCPC may prevent damage caused by AFB1 in broilers by inhibiting kidney oxidative stress and cell apoptosis (22). Mitochondrial injury is closely related to several diseases. Induction of autophagy increases the threshold for cell death and inhibits apoptosis. Autophagy can protect kidney cell mitochondria from damages and maintain cellular bioenergetics. However, the most advantageous approach for alleviating the toxic effects of AFB1 on kidney cells of chickens with traditional Chinese medicine has not been reported. Therefore, the aim of this study was to explore the mitochondrial autophagy to understand the underlying mechanism of AFB1 causing kidney apoptosis and drugs exertion effects. In this study, kidneys of chickens were selected to investigate the toxic effects of AFB1 and explore the efficacy of PCPC for alleviating mycotoxin-mediated toxicity.

## Materials and Methods

### Preparation of PCPC and Positive Drug YCHT (Yin-Chen-Hao Tang) Extract

#### Preparation of PCPC

PCPC (20 g *P*. *chinense* Pursh, 20 g *Bupleurum*, 4 g Yinchen, and 4 g licorice; Chongqing, Renjihong Pharmaceutical Co., Ltd.) was added to 480 ml deionized water, after which the volatile oil was extracted and collected at 140°C following filtration. Subsequently, 480 ml deionized water was added to the residue at 140°C, following decoction for 1 h which was filtered, and 480 ml of deionized water was added again to decoct for 1 h. The solution was then combined with the three water extracts. Next, 3.84 l of 60% ethanol was added to the water-extracted residue and extracted for 10 min under ultrasonication at 125 W and 50°C. The volatile oil, water, and alcohol extracts were concentrated in a rotary evaporator to 48 ml (equivalent to 1 g herb ml^−1^) and then stored at 4°C.

#### Preparation of YCHT Extract

YCHT was prepared according to the description in “ShanghanLun” (23). First, 54 g Yin Chen was decocted using 3.6 L deionized water, and the solution volume was reduced to 1.8 L, after which samples of 27 g gardenia and 18 g rhubarb were added. The mixture was decocted for 30 min and filtered through a bandage. Subsequently, the filtrate was concentrated under vacuum to 100 ml (equivalent to 1 g herb ml^−1^) and stored at 4°C.

### Experimental Design, Birds, and Sample Collection

A total of 180 Cobb broilers (age, 1 day) were purchased from a commercial hatchery (Daan, Zigong, Sichuan, China; License No. 1510304017011579). All experimental protocols were performed in accordance with animal ethics guidelines and approved by the Institutional Animal Care and Use Committee (IACUC) of Southwest University (IACUC-20201203). All birds were maintained under standard hygienic conditions, ambient temperature was set at 33°C and 60% humidity during the experimental period. After 1 week of adaptive feeding, they were randomly assigned to the following groups (*n* = 30 per group): control; AFB1; positive drug (YCHT group); and PCPC high-, PCPC medium-, and PCPC low-dose groups. All groups were fed AFB1 (MACKLIN, Shanghai, China) at 2.8 mg/kg feed except for the control group; the positive drug group was fed YCHT at 10 ml/kg feed, and the PCPC high-, medium-, and low-dose groups were fed normal feed containing 15, 10, and 5 ml/kg PCPC until the end of the experiment (28 days), respectively. All birds had free access to feed; diet and body growth parameters were recorded during each week of the experiment, and serum and kidney samples were collected on the 28th day of the experiment. The birds were euthanized without anesthesia via cervical dislocation; all birds were weighed, and kidney index was calculated using the following formula:


$$\begin{array}{l} \text{Organ index}=\frac{\text{organ mass }\left(g\right)}{\text{body weight }\left(100\;g\right)} \end{array}$$

### Histopathological and Serum Analyses

Kidney samples were stained using hematoxylin and eosin (H&E; Solar Bio, Beijing, China). In brief, part of the broiler kidney tissue was fixed in 10% formaldehyde solution for more than 24 h; placed in running water overnight; and then the kidney sample was dehydrated in a series of ethanol, turned transparent in xylene, and embedded in paraffin. The tissue samples were cut into 5-μm sections, placed on glass slides, stained with H&E, sealed with neutral resin, and the pathological changes in the kidney tissues were observed under an optical microscope (Zeiss upright microscope Axio Scope A1; Carl Zeiss, Oberkochen, Germany). Serum urea and uric acid (UA) levels in broilers were analyzed using an automatic biochemical analyzer.

### Oxidative and Autophagy Assay

Kidneys samples (total six samples) were collected and immediately frozen in liquid nitrogen at −80°C. The kidney (*n* = 6) samples were added to phosphate-buffered saline (pH 7.4) (kidney and PBS volume 1:9), fully homogenized using a tissue homogenizer, and centrifuged (4°C, 3,000 rpm for 20 min). The superoxide dismutase (SOD), malondialdehyde (MDA), Beclin1, LC3-I, and LC3-II levels in the kidney tissues were analyzed using a commercial kit according to the manufacturer's instructions (Xiamen Huijia Biological Technology Co., Ltd., Xiamen, China).

### RNA Isolation and Quantitative Real-Time Reverse Transcription-Polymerase Chain Reaction (RT-qPCR)

Kidney tissue samples were collected from each group, ground in liquid nitrogen, and then homogenized in TRIzol reagent (1 ml trizol for 50 mg kidney sample) (Win Biosciences, Beijing, China) to extract the total RNA. cDNA was synthesized from the RNA using a Trans Gen cDNA kit (Biotech Co., Ltd., Beijing, China) according to the manufacturer's instructions. Reverse transcription was performed at 42°C for 60 min and 95°C for 3 min; cDNA synthesis was performed in a 20-μl reaction mixture consisting of oligo (dT)18, 2 × ES Reaction Mix, and 5 μg RNA.

The mRNA expression of genes encoding nuclear factor erythroid 2 (*NRF2*), heme oxygenase-1 (*HMOX1*), B-cell lymphoma 2 (*BCL2*), P53 (*P53*), BAX (*BAX)*, BAK (*BAK1*), cytochrome *c* (*CYC*), caspase-9 (*CASP9*), and caspase-3 (*CASP3*) was analyzed via SYBR Green I real-time fluorescent quantitative PCR (relative quantification). The primer sequences of the genes are shown in Table 1. All PCR reactions were run at least in quadruplicate using the TransStart Green qPCR SuperMix kit (TransGen Biotech, Beijing, China) in a 20-μl reaction volume consisting of 2 μl cDNA, specific forward and reverse primers (0.5 μl each), and 10 μl Green qPCR Super Mix. The following thermal cycling parameters were used: 94°C for 30 s, 40 amplification cycles at 94°C for 5 s, 61°C for 35 s, and 72°C for 30 s. Relative quantification of each gene was performed using the comparative 2^−Δ*ΔCT*^ method (24, 25).

**Table 1:** Primers for quantitative real-time PCR.

**Target gene**	**Primer sequence (5^**′**^-3^**′**^)**	**Product length (bp)**
Nrf-2	F: GCATTTTGCAGCCAGACGAC	20
	R: TTGTTCCTGTGTCACCGTCC	20
HO-1	F: CAACGCCACCAAGTTCAAACA	21
	R: CAGCGCCTCAAACACCTGTA	20
Bcl-2	F: CTGGATCCAGGACAACGGA	19
	R: GATGCAAGCTCCCACCAGAA	20
P53	F: GTCCCATCCACGGAGGATTAT	21
	R: CCAGGCGGCAATAGACCTTA	20
Bax	F: CAGATTGGAGAGGCCCTCTT	20
	R: AATCTGGTCCTGGCTGTTGC	20
Bak	F: GGCCATCACGAGAGATCAATG	21
	R: TCCTGTTGGTAGCGGTAGAAG	21
Cyt-c	F:CCCAGTGCCATACGGTTGAA	20
	R:GCTTGTCCTGTTTTGCGTCC	20
Caspase-9	F: CCGGAGGGATTTATGGAACAG	21
	R: CAGGCCTGGATGAAGAAGAGT	21
Caspase-3	F: GAAGATCACAGCAAGCGAAGC	21
	R: CAAGAGGGCCATCTGTACCAT	21
GAPDH	F: CAGAACATCATCCCAGCGTC	20
	R: GGCAGGTCAGGTCAACAAC	19

### Statistical Analysis

Experimental data were analyzed by one-way ANOVA using SPSS 20.0.0 (IBM, Armonk, NY, USA) software. Results were presented as mean ± SD. The significant difference among treatments was determined using Duncan's multiple-range test. Graph Pad Prism version 8.0.1 (Graph Pad Software, San Diego, CA, USA) was used to generate graphs with error bars.

## Results

### Effect of PCPC Treatment on Weight Gain and Growth Performance

**Figure 1:**
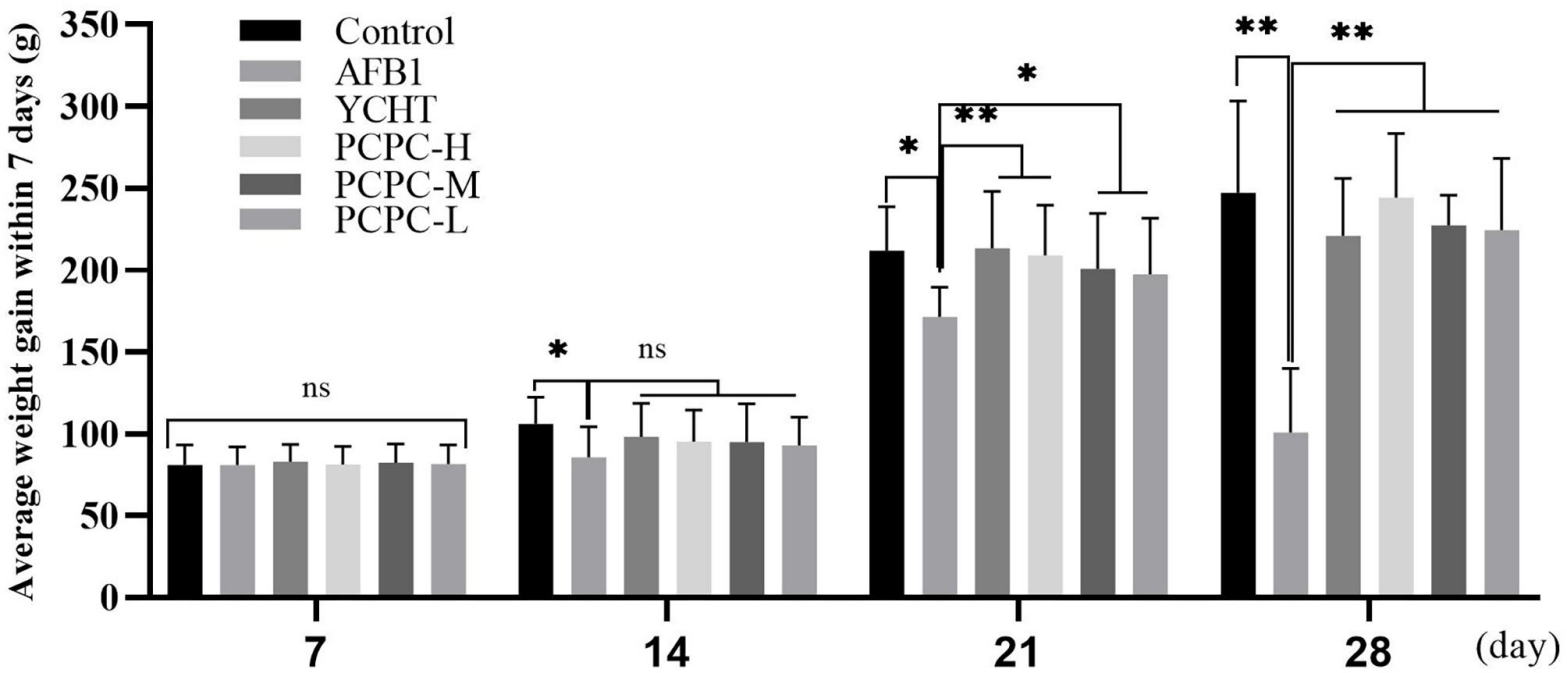
Effect of PCPC on weight gain and growth performance of broilers fed diet containing AFB1. The average weekly body weight of each group. Asterisk means values within columns are significantly different (*p* < 0.05) (ns, non-significant). *p < 0.05 and **p < 0.01.

The effect of PCPC treatment on weekly growth performance (days 7, 14, 21, and 28) is summarized in Figure 1. The average body weight of each group increased within the first 7 days; however, the difference between the groups was not significant (*p* > 0.05). The group of chickens (age, 2–4 weeks) fed AFB1 showed a lower average weekly weight gain than that of other groups. Supplementation with PCPC and positive drug alleviated this adverse effect, with a significant increase in weekly weight gain and performance of broilers compared to that of the AFB1-fed group (Figure 1).

### Biochemical Analysis of Serum Urea and UA Levels, and Kidney Index

**Figure 2:**
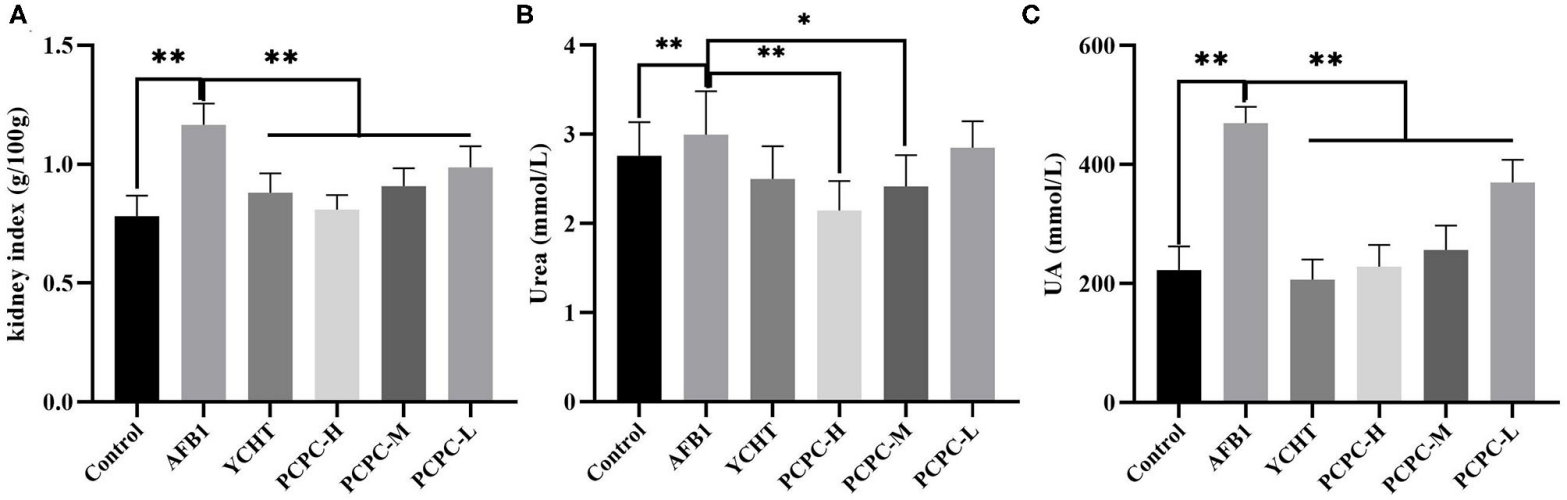
Biochemical analysis of serum urea and UA levels and the kidney index analyzed on the 28th day of the experiment. **(A)** Kidney index; **(B)** Urea; **(C)** UA. Asterisk means values within columns are significantly different. *p < 0.05 and **p < 0.01.

To evaluate the effect of AFB1 and PCPC treatment on kidney function, serum urea and UA levels and the kidney index were analyzed on the 28th day of the experiment. AFB1 treatment resulted in increased the kidney index and serum urea and UA levels compared to those in the control group (*p* < 0.05). PCPC and positive drug supplementation significantly ameliorated these adverse effects by decreasing the kidney index and serum urea and UA levels compared to those in the AFB1 group (Figure 2).

### Histopathological Analysis of Kidney Tissues

**Figure 3:**
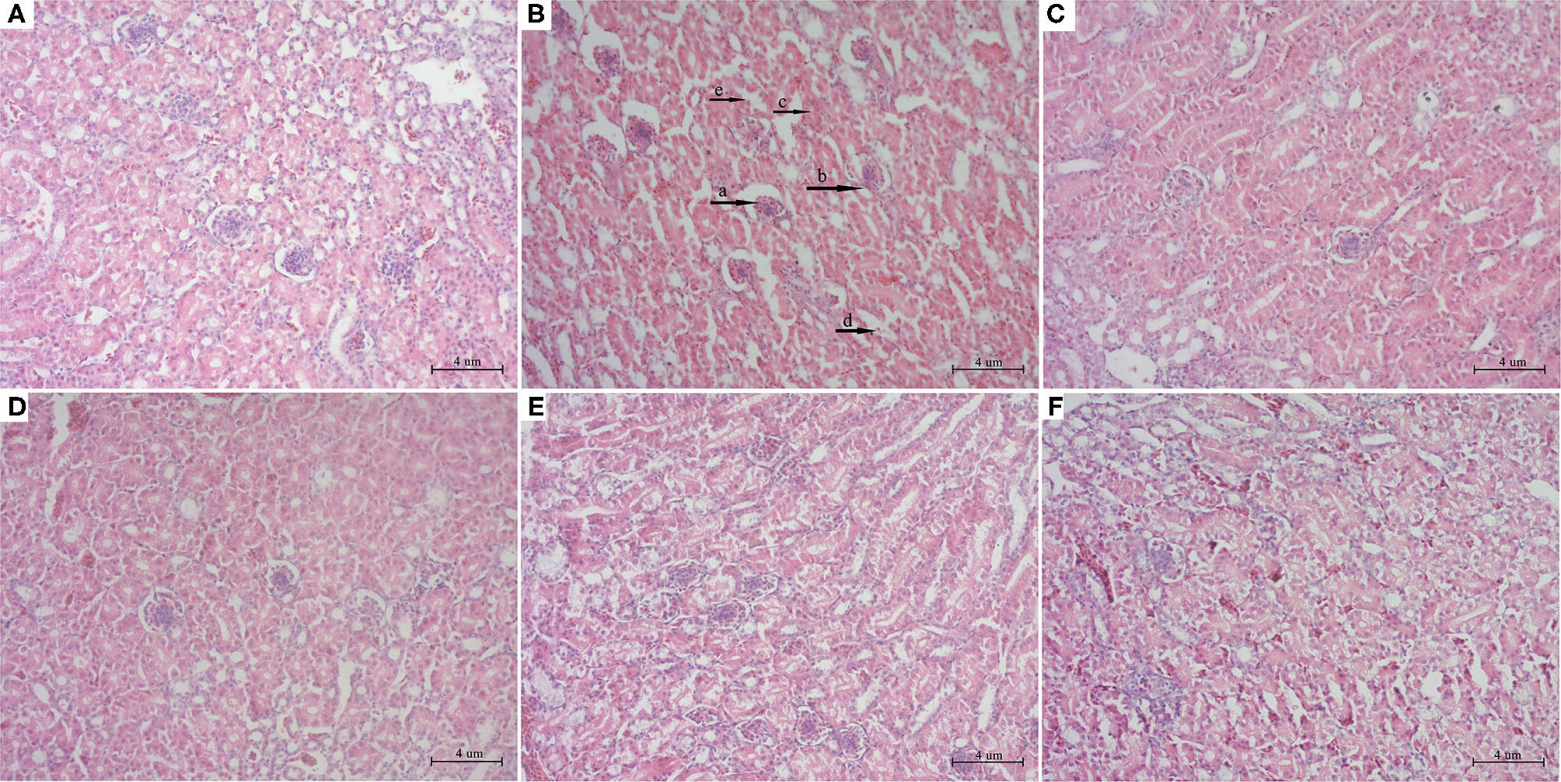
Histopathological analysis of kidney from different groups. The kidney tissues were stained with H&E stain. **(A)** Control; **(B)** AFB1; **(C)** YCHT; **(D)** PCPC high dose; **(E)** PCPC medium dose; **(F)** PCPC low dose. (a) glomerulus atrophy, (b) glomerular epithelial cell proliferation, (c) renal tubular lumen stenosis and obscurity, (d) renal tubular epithelial cell shedding.

To further explore the effects of PCPC and AFB1 treatment on kidney tissues, H&E staining was performed. Results showed that AFB1 supplementation resulted in kidney tissue injury compared to that in the control group; tissue sections of the AFB1-treated group showed edema and inflammatory cells with glomerulus atrophy, glomerular epithelial cell proliferation, renal tubular lumen stenosis and obscurity, and renal tubular epithelial cell shedding. However, PCPC and positive drug supplementation significantly reduced the development of abnormal histological signs, renal injuries, and structural deterioration in the kidney tissues compared to those in the AFB1 group (Figure 3).

### Serum Antioxidant Parameters

**Figure 4:**
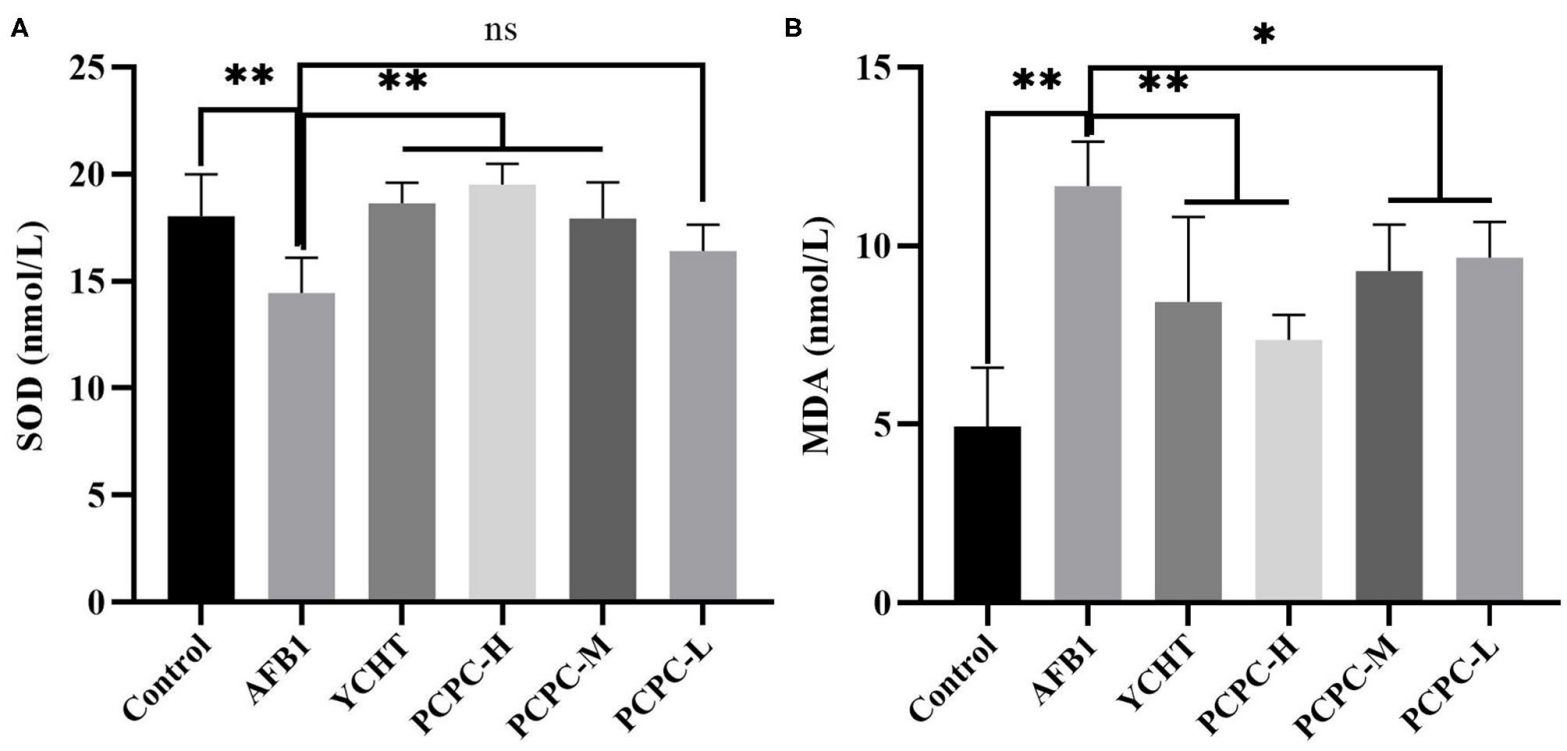
Effect of PCPC on serum antioxidant parameters of broiler fed diet containing AFB1. Values are represented as the mean ± SD. **(A)** SOD; **(B)** MDA. Mean values within a column are significantly different (*p* < 0.05). *p < 0.05 and **p < 0.01, ns, non-significant.

The effect of PCPC treatment on serum antioxidant indices of broiler kidneys was evaluated. We found that the SOD level decreased and MDA level increased significantly in the AFB1 group (*p* < 0.01) compared to those in the control group. Administration of PCPC and positive drug to AFB1-treated groups significantly enhanced the antioxidant enzyme activities in a dose-dependent manner by increasing SOD levels and decreasing MDA levels compared to those in AFB1-treated birds (Figure 4).

### Expression Levels of *NRF2* and *HMOX1* in Kidneys

**Figure 5:**
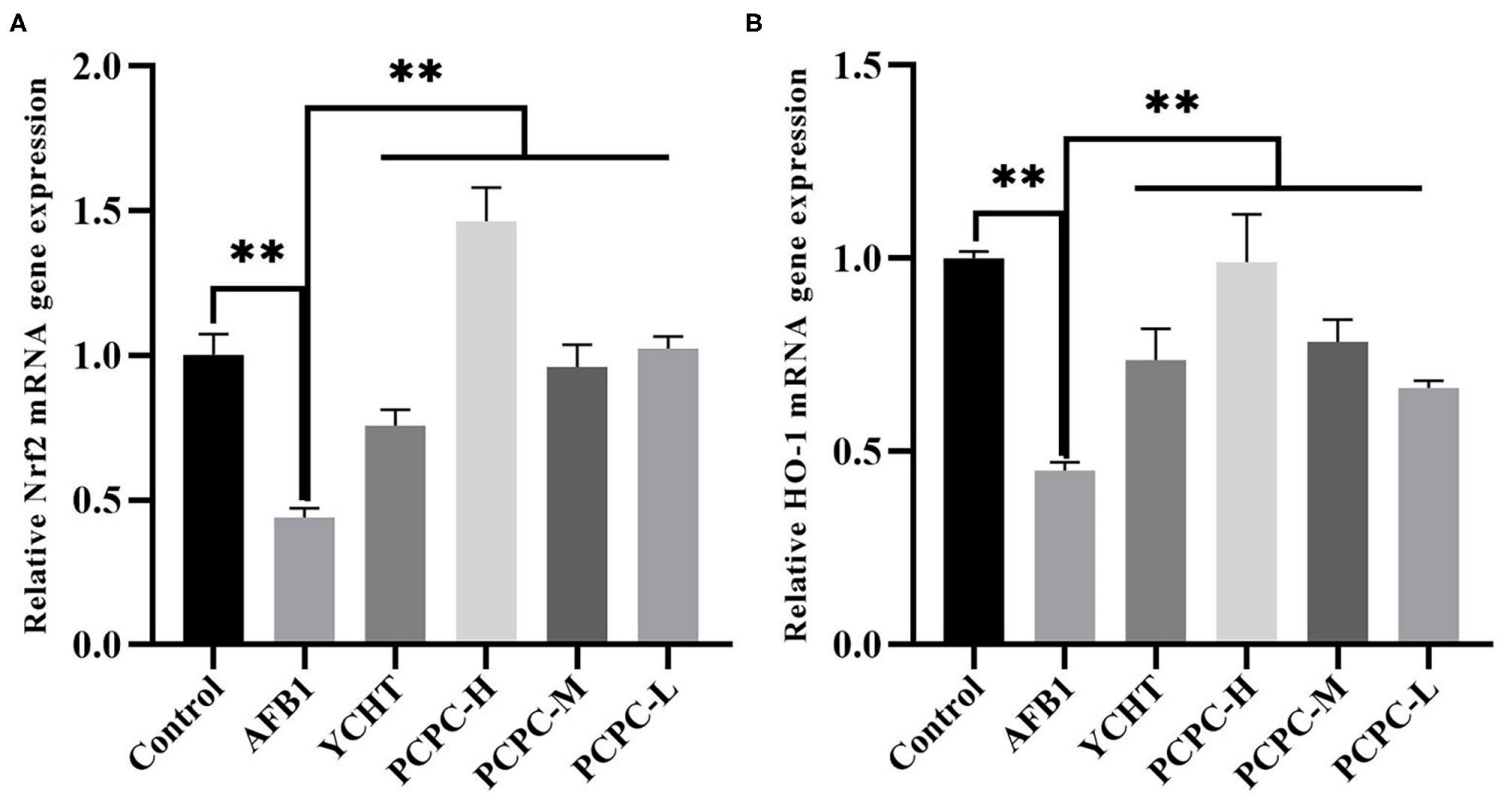
Effect of PCPC on expression levels of Nrf2 and HO-1 in kidney of broilers. The mRNA expression of Nrf2 and HO-1 was identified by quantitative real-time PCR. All data are expressed as means ± SD. **(A)** Nrf2 expression; **(B)** HO-1 expression. Asterisk indicates significant difference (p < 0.05) compared to the control group and AFB1 group.

The mRNA expression levels of *NRF2* and *HMOX1* in the kidneys of broilers in the AFB1 group were significantly reduced (*p* < 0.01) compared to those in the control group. In contrast, the *NRF2* and *HMOX1* mRNA levels in the PCPC and positive drug treatment groups were significantly increased (*p* < 0.01; Figure 5).

### Effect of PCPC and AFB1 Treatment on Apoptosis in the Kidney Tissues

**Figure 6:**
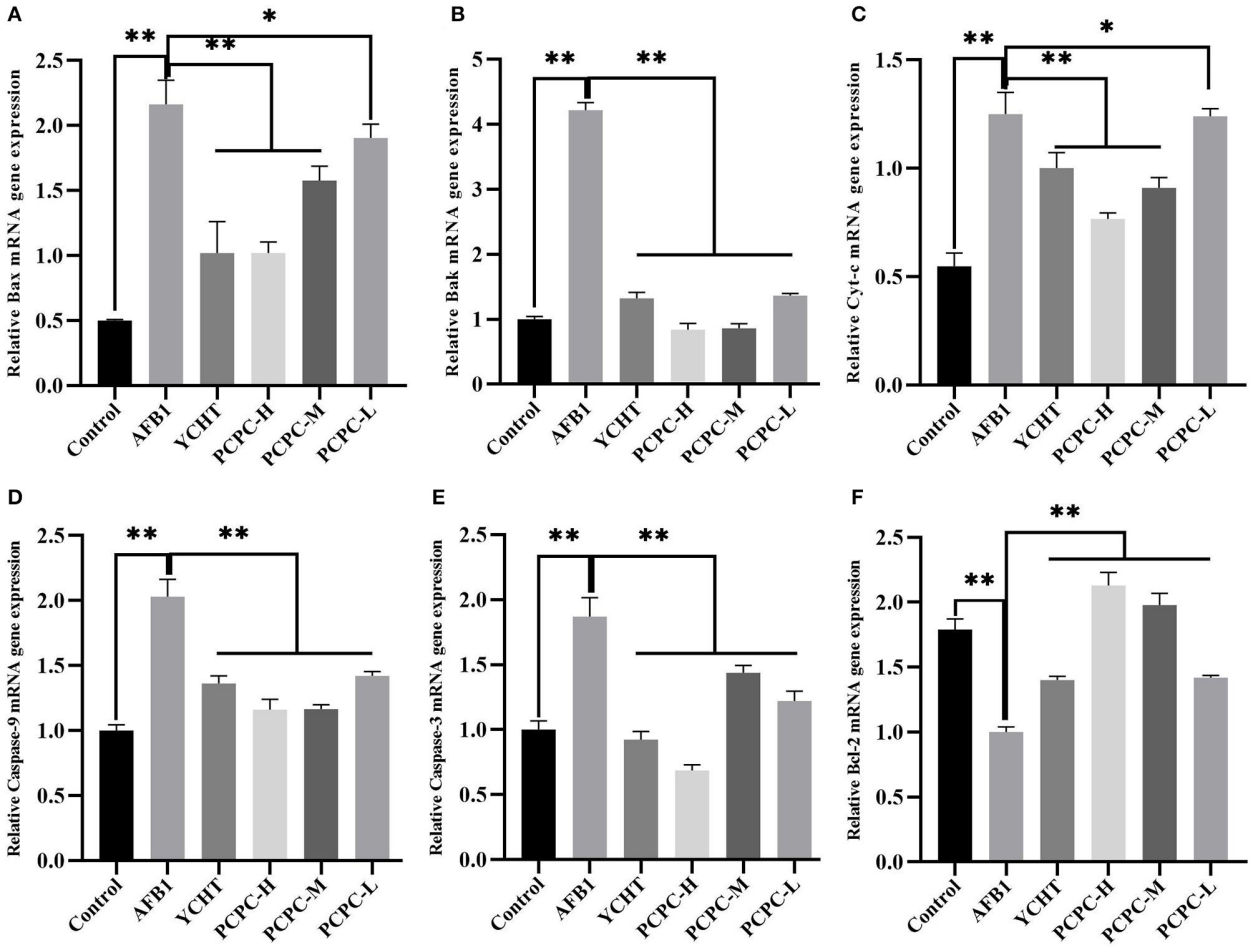
Effect of PCPC and AFB_1_ on apoptosis-related genes in the kidney of broilers. The mRNA expression of apoptosis-related genes were identified by quantitative real-time PCR. All data are expressed as means ± SD. **(A)** Bax; **(B)** Bak; **(C)** Cyt-c; **(D)** Caspase-9; **(E)** Caspase-3; **(F)** Bcl-2. Asterisk indicates significant difference (*p* < 0.05) compared to the control group and AFB1 group. *p < 0.05 and **p < 0.01.

The mRNA levels of mitochondrial apoptosis-related genes were analyzed in the kidney tissues of chickens to evaluate the ameliorative effects of PCPC against AFB1-mediated injuries and pathogenesis. The mRNA expression level of *BCL2* decreased and the levels of *BAX, BAK1, CYC, CASP9*, and *CASP3* increased in the AFB1-treated groups compared to those in the control group (*p* < 0.01). However, the change in expression levels of these mitochondrial apoptosis-related genes was significantly (*p* < 0.01) reversed in the PCPC and positive drug treatment groups compared to the trend in the AFB1-treated group (Figure 6).

### Effect of PCPC and AFB1 Treatment on Autophagy in Kidney Tissues

**Figure 7:**
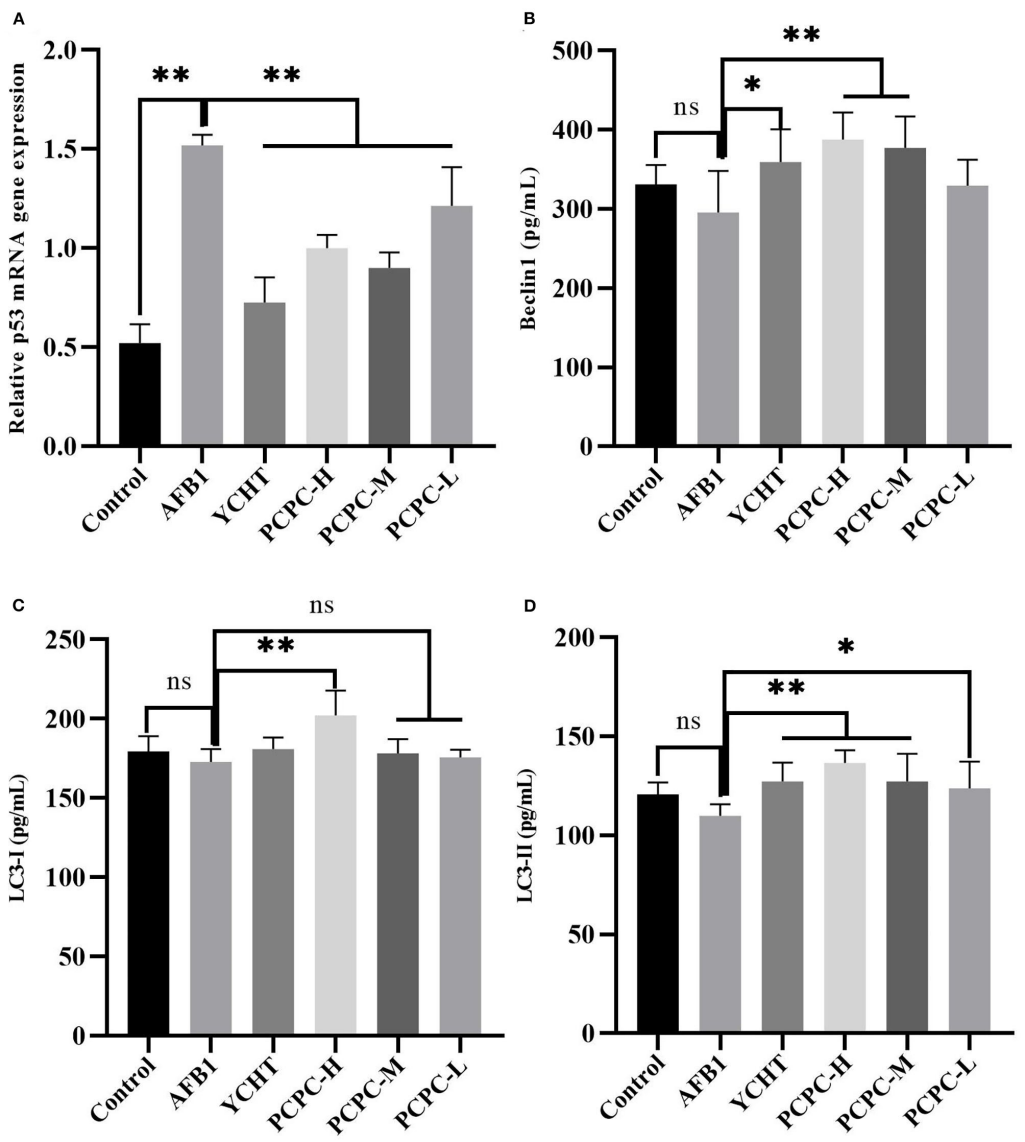
Effect of PCPC and AFB_1_ on autophagy-related genes (Beclin1, LC3-I and LC3-II, and p53) in kidneys. All data are expressed as mean ± SD. **(A)** p53; **(B)** Beclin1; **(C)** LC3-I; **(D)** LC3-II. Asterisk indicates significant difference (*p* < 0.05) compared to the control and AFB1 group. *p < 0.05 and **p < 0.01, ns, non-significant.

AFB1 treatment downregulated Beclin1, LC3-I, and LC3-II, and upregulated p53 compared to those in the control group. PCPC and positive drug treatment significantly reversed these changes in mRNA levels of autophagy-related genes compared to those in the AFB1-treated group (*p* < 0.05; Figure 7).

## Discussion

Birds are susceptible to AFB1-mediated toxicity (26). Exposure to AFB1 can cause oxidative stress, immunosuppression, inflammation, and cell apoptosis, and it affects many physiological processes in birds (27). Moreover, due to the ubiquity of AFB1 in the environment, it represents a significant threat to the aquaculture industry and animal product safety (26). The present study evaluated the protective effect of PCPC against AFB1-induced kidney toxicity in broilers and determined the mitochondrial apoptosis-related pathways associated with PCPC and AFB1 treatments.

AFB1 can reduce the growth rate of broilers, leading to higher morbidity and mortality (28, 29). In the current study, we similarly found that the growth performance of broilers significantly decreased with the continuous intake of AFB1, whereas the growth performance of broilers was improved after adding PCPC to the feed. The kidneys are the main organ associated with the excretion and accumulation of AFB1; therefore, AFB1 can disrupt the structure of the kidney and cause organ damage (30). Urea and UA levels in the blood represent major indicators of pathological changes and kidney function (31). The organ index is an indicator of organ toxicity (32). Our findings showed an abnormal increase in kidney index and urea and UA levels; however, these alterations reversed upon PCPC supplementation. The glomerulus and tubules constitute the functional unit (nephron) of the kidney. The glomerulus is a spheroid composed of capillaries which filter the blood (33). The glomerular basement membrane is crucial to the glomerular capillaries and is a necessary structure required for the kidneys to perform filtration (34). The renal tubules are responsible for a variety of transport processes and they secrete or reabsorb electrolytes and metabolites in urine (35). We observed glomeruli atrophy in kidney tissue sections; the glomerular basement membrane proliferated and the renal tubules fell off owing to AFB1-mediated injury, indicating that the kidney filtration functions were impaired; however, kidney function significantly improved with PCPC treatment.

Oxidative stress represents one of the main pathogenesis mechanisms of several diseases (36). It promotes the development of diseases by affecting redox homeostasis in the body. Renal insufficiency is generally believed to be related to an imbalance in redox homeostasis (37). AFB1 can induce the generation of high levels of free radicals and promote lipid peroxidation, thereby increasing oxidative stress in the kidneys (38). SOD and MDA are the most important elements related to oxidative stress. SOD is an antioxidant enzyme that plays a vital role in removing O^2−^ (39). MDA is an oxidized lipid metabolite that serves as an indicator of cellular damage and lipid peroxidation status (40). In the present study, the addition of AFB1 to the feed induced a significant increase in MDA levels and decreased SOD levels in the kidneys of broilers; therefore, AFB1 treatment induced oxidative stress generation in the kidneys of broilers. PCPC treatment inhibited the accumulation of MDA and increased the level of SOD, indicating that PCPC can alleviate oxidative stress production in the kidneys induced by AFB1. *Penthorum chinense* Pursh, usually used as a traditional medicine, is studied for its therapeutic effects in diuresis, detoxification, and blood circulation promotion. In addition, the study concluded that PCP herb demonstrated optimistic effects in anti-oxidation, anti-hepatitis, and anti-tumor efficacy (41). Our results are consistent with those of previous studies which revealed that AFB1 can induce oxidative stress in the kidneys of broilers (26).

NRF2 functions as a regulator of the intracellular redox state, and it directly regulates the antioxidant defense system via various mechanisms (42). HMOX1, an important downstream antioxidant enzyme in the NRF2 signaling pathway, has the ability to enhance antioxidant activity (43, 44). In this study, we investigated the mechanism by which AFB1 induces oxidative stress by measuring the mRNA expression levels of *NRF2* and *HMOX1* in the NRF2 signaling pathway. The findings revealed that *NRF2* and *HMOX1* were downregulated in the kidney tissues of the AFB1-treated group, and PCPC treatment upregulated these genes and regulated the NRF2 signaling pathway to protect the kidney from oxidative stress, thereby treating kidney damage caused by AFB1 (45).

Apoptosis involves programmed cell death that can effectively remove damaged cells and is essential for animal development (46, 47). However, excessive apoptosis can cause organ damage and is considered to be one of the mechanisms of AFB1-induced toxicity (48–50). Apoptosis may occur via two different apoptotic pathways, the extrinsic and intrinsic apoptotic pathways, which are triggered by internal mitochondria-mediated signal transduction or external receptor-dependent stimuli (46, 51). Mitochondria are essential organelles required for maintaining the viability of eukaryotic cells, and the role of mitochondria in apoptosis has been established (52, 53). The tumor suppressor P53 interacts with members of the BCL2 family present on the mitochondrial outer membrane; P53 can bind to BAK and catalyze BAK activation, and promote transcription-independent activation of BAX. Subsequently, the activated BAK and BAX oligomerize in the mitochondrial outer membrane to induce permeabilization. Proapoptotic factors (such as CYC) are released from the mitochondria into the cytosol to activate the caspase cascade via this pathway (54, 55). Therefore, internal mitochondria-mediated signal transduction activates caspase-9 and leads to the activation of caspase-3 to promote apoptosis. In contrast, BCL2 inhibits the activation of BAX and BAK, thus inhibiting apoptosis (56). In our study, we mainly explored the effect of AFB1 and PCPC on the mitochondria-mediated apoptosis pathway. AFB1 inhibited the transcription of *BCL2* in the kidney tissues of broilers and promoted the transcription of *P53, BAX, BAK1, CYC, CASP9*, and *CASP3*, and induced kidney cell apoptosis via the mitochondrial pathway. These findings indicate that AFB1 may activate the mitochondria-mediated apoptosis pathway (57). However, PCPC treatment inhibited the overexpression of P53, BAX, BAK, CYC, CASP9, and CASP3 induced by AFB1, whereas upregulation of BCL2 was observed. Therefore, PCPC treatment protected kidney cells from excessive apoptosis by inhibiting the mitochondrial apoptosis pathway activated by AFB1.

Autophagy is a highly conserved catabolic process that maintains homeostasis under adverse conditions (58, 59). It is generally agreed that autophagy is closely related to apoptosis, and induction of autophagy increases the threshold for cell death and inhibits apoptosis to reduce cytotoxicity (60). Unc-51-like autophagy activating kinase 1 (ULK1) and class III phosphatidylinositol 3-kinase complex I (PI3KC3-C1) are important proteins that initiate autophagy (61). Beclin1 is a part of PI3KC3-C1 and mediates the formation of a phagophore and functions as an autophagy initiation factor via interaction with PtdIns(3)-kinase (62). In the dynamic process of autophagosome formation, ubiquitin-like enzymatic cascades function after ULK1 and PI3KC3-C1 activity; thus, cytosolic LC3-I conjugates to phosphatidyl ethanol amine to form the autophagic marker LC3 (63). In this study, the expression levels of autophagy-associated genes were decreased in the AFB1 group, thereby inhibiting autophagy. Our findings showed that mitochondrial function damage and autophagy-related indicators were related to chicken kidney damage, oxidative injury, and pathogenesis induced by AFB1.

## Conclusion

The addition of PCPC to an AFB1-contaminated diet had a positive effect on growth and performance and reduced AFB1 pathogenesis and degenerative changes in the kidneys; PCPC treatment activated autophagy and regulated abnormal kidney function and the imbalance in mitochondrial dynamics. Therefore, PCPC effectively prevented oxidative stress and apoptosis in infected broiler chickens supplemented with AFB1. PCPC represents a natural and safe agent for treating avian mycotoxin-mediated toxicity.

## Data Availability Statement

The original contributions presented in the study are included in the article/supplementary files, further inquiries can be directed to the corresponding author/s.

## Ethics Statement

The experimental protocols were performed in accordance with animal ethics guidelines and reviewed and approved by the Institutional Animal Care and Use Committee (IACUC) of Southwest University (IACUC-20201203).

## Author Contributions

WT, FN, and JL conception and designed of the study. WT and JL analyzed and interpretation for the data. WT, ZL, YH, and ZH performed the experiment. WT and FN wrote the first draft of manuscript. All authors contributed to manuscript revision and read and approved the submitted version.

## Funding

This research was funded by Special Project for Fundamental Work of Science and Technology; Grant No. 2013FY110600-03 and special funding for Chongqing Post-Doctoral Research project 2020 number 7820100603.

## Conflict of Interest

The authors declare that the research was conducted in the absence of any commercial or financial relationships that could be construed as a potential conflict of interest. The handling editor declared a past co-authorship with one of the authors, FN.

## Publisher's Note

All claims expressed in this article are solely those of the authors and do not necessarily represent those of their affiliated organizations, or those of the publisher, the editors and the reviewers. Any product that may be evaluated in this article, or claim that may be made by its manufacturer, is not guaranteed or endorsed by the publisher.
